# Hand Motion Analysis Using Accelerometer-Based Sensors and Sheep’s Head Model for Basic Training in Functional Endoscopic Sinus Surgery

**DOI:** 10.7759/cureus.59725

**Published:** 2024-05-06

**Authors:** Constantin Stan, Peter L Ujvary, Cristina Blebea, Mihai I Tănase, Mara Tănase, Septimiu Sever Pop, Alma A Maniu, Marcel Cosgarea, Doinel G Rădeanu

**Affiliations:** 1 Otolaryngology, Iuliu Hațieganu University of Medicine and Pharmacy, Cluj-Napoca, ROU; 2 Surgical Clinical, Faculty of Medicine and Pharmacy, "Dunarea de Jos" University of Galati, Galati, ROU; 3 Otolaryngology, Iuliu Hațieganu University of Medicine and Pharmacy, Cluj Napoca, ROU

**Keywords:** hand motion analysis, simulation in medical education, sheep's head, functional endoscopic sinus surgery training, accelerometer

## Abstract

Introduction: Motion analysis, the study of movement patterns to evaluate performance, plays a crucial role in surgical training. It provides objective data that can be used to assess and improve trainee's precision, efficiency, and overall surgical technique. The primary aim of this study is to employ accelerometer-based sensors placed on the wrist to analyze hand motions during endoscopic sinus surgery training using the sheep's head. By capturing detailed movement data, the study seeks to quantify the motion characteristics that distinguish different levels of surgical expertise. This approach seeks to quantify motion characteristics indicative of surgical expertise and enhance the objectivity and effectiveness of surgical training feedback mechanisms.

Materials and methods: Twenty-four participants were divided into three groups based on their experience with endoscopic endonasal surgery. Each participant was tasked with performing specified procedures on an individual sheep's head, concentrating on exploring both nasal passages. A single Bluetooth Accelerometer WitMotion sensor was mounted on the dorsal surface of each hand. This facilitates the evaluation of efficiency parameters such as time, path length, and acceleration during the training procedures. Accelerometer data were collected and imported in CSV format (comma-separated values) for each group of surgeons-senior, specialist, and resident-mean values and standard deviations were computed. The Shapiro-Wilk Test assessed the normality of the distribution. The Kruskal-Wallis test was employed to compare procedural time, acceleration, and path length differences across the three surgeon experience levels.

Results:* *For the procedural time, statistical significance appears in all surgical steps (p<0.001), with the biggest difference in the septoplasty group in favor of the senior group. A clear difference can be observed between the resulting acceleration of the dominant hands (instrument hand) and the non-dominant hand (endoscopic hand) and between the study groups. The difference between groups reaches statistical significance with a p-value <0.001. A statistically significant difference can be seen between the paths covered by each hand of every participant (p<0.001). Also, senior doctors covered significantly less movement with both hands than the specialists and the resident doctors (p<0.001).

Conclusions: The data show a clear learning curve from resident to senior, with residents taking more time and using more hand movements to complete the same tasks. Specialists are in the intermediate phase, showing signs of honing their technique towards efficiency. This comprehensive data set can help tailor training programs to focus on both efficiency (quicker procedures) and economy of motion (reduced path length and acceleration), especially in more complex procedures where the difference in performance is more pronounced.

## Introduction

The field of surgical training has continually evolved to incorporate advanced technologies that enhance the learning experience and improve skill acquisition. Motion analysis, the study of movement patterns to evaluate performance, plays a crucial role in surgical training. It provides objective data that can be used to assess and improve trainees' precision, efficiency, and overall surgical technique. In particular, the complex and delicate nature of endoscopic sinus surgery, with its reliance on fine motor skills and spatial awareness, makes motion analysis an invaluable tool for training programs.

Motion analysis is pivotal in surgical training, objectively measuring a surgeon's dexterity and proficiency. Moorthy et al. emphasized that dexterity is crucial to surgical competence, particularly in minimally invasive surgery. Their work with the Imperial College Surgical Assessment Device (ICSAD) demonstrated its effectiveness in distinguishing surgeons' skills based on motion analysis, highlighting its potential as a valuable training tool [1].

Assessing surgical skills presents significant challenges, especially for intricate procedures like endoscopic sinus surgery. Traditional assessment methods often rely on subjective evaluations or outcome-based metrics, which may not fully capture a surgeon's technical proficiency or the nuances of their hand movements. This limitation is compounded by the high stakes of surgical performance, where precision is critical to patient outcomes. Furthermore, there is a need for objective, quantifiable measures of surgical skill that can provide consistent feedback for trainees, helping to identify specific areas for improvement. Mason et al. have shown that motion analysis can serve as a valid tool for assessing laparoscopic skills, suggesting its applicability in broader surgical contexts, including endoscopic sinus surgery, to overcome these assessment challenges [2].

The primary aim of this study is to employ accelerometer-based sensors placed on the wrist to analyze hand motions during endoscopic sinus surgery training. By capturing detailed movement data, the study seeks to quantify the motion characteristics that distinguish different levels of surgical expertise. This approach seeks to quantify motion characteristics indicative of surgical expertise and enhance the objectivity and effectiveness of surgical training feedback mechanisms.

To evaluate the precision and efficiency of surgical maneuvers, an array of measurable parameters were used, including the total time taken to complete a specific surgical action, the average rate of acceleration for both the dominant and the non-dominant hand, and the comprehensive distance traveled by the surgical instrument while performing the operative steps. Additionally, the measurement of the path length directly aligned with the axis of the instrument provides further insight into the surgical technique's accuracy.

These measured parameters are useful in identifying the factors of failure and success in acquiring surgical skills and differentiating a beginner from an advanced practitioner [3]. Measurements were conducted using the sheep's head as an alternative training model in functional endoscopic sinus surgery, to which we referred and analyzed in detail in our previous articles [4,5].

## Materials and methods

Participants

The inclusion criteria for participants in the study were based on their level of experience in endoscopic sinus surgery and their affiliation with our training center. Consequently, the participants were divided into three distinct groups according to their experience in endoscopic endonasal surgery. The first group was formed by ten first-year otolaryngology residents with little or no experience in endoscopic endonasal surgical procedures. The second group comprised ten junior otolaryngology specialists with work experience between 3 to 5 years in rhinologic procedures. Finally, the third group consisted of 4 senior otolaryngologists whose experience was 20 years or more.

Procedures and equipment

Twenty-four fresh-frozen adult sheep heads (Native Romanian Turcana sheep) were procured from the local slaughterhouse with veterinary clearance obtained. A preparatory phase preceded the start of these tasks, during which the fresh frozen sheep heads were gradually defrosted at ambient temperature for about 14 hours. Following this, the sheep heads' nasal passages were flushed with saline as part of the preparation. Then, to ease the process of endoscopic navigation, the nose's tip was cut before the assessment began. Each participant was assigned the task of performing the specified procedures on an individual sheep's head, focusing on exploring both nasal passages. Each was given a sheep head for this purpose.

All participants received theoretical instruction on the procedures they were to perform in the earlier part of the study, so this time, they will repeat the same five maneuvers using the same equipment and tools (Table 1).

**Table 1:** The list of equipment and tools used in the training.

Nr	Equipment (Germany—KARL STORZ SE & Co. KG, Tuttlingen)
1.	Karl Storz TelePack X
2.	Karl Storz rigid Hopkins telescopes 0° and 30°
3.	Mladina head holder (Karl Storz^®^)
4.	Blakesley nasal forceps (Karl Storz^®^)
5.	Backbiter antrum punch (Karl Storz^®^)
6.	Cottle dorsal scissors (Karl Storz^®^)
7.	Freer elevator (Karl Storz^®^)
8.	Sickle knife (Karl Storz^®^)
9.	Straight and curved curette (Karl Storz^®^)
10.	Frazier suction tube (Karl Storz^®^)
11.	Scalpel handle with no. 11 blade (Karl Storz^®^)

The surgical procedures performed included the endoscopic extraction of foreign objects like spherical plastics and popcorn, the endoscopic septoplasty, the endoscopic turbinoplasty, the maxillary anthrostomy, and the endoscopic removal of the ethmoid sinus cells.

Motion sensors

**Figure 1:**
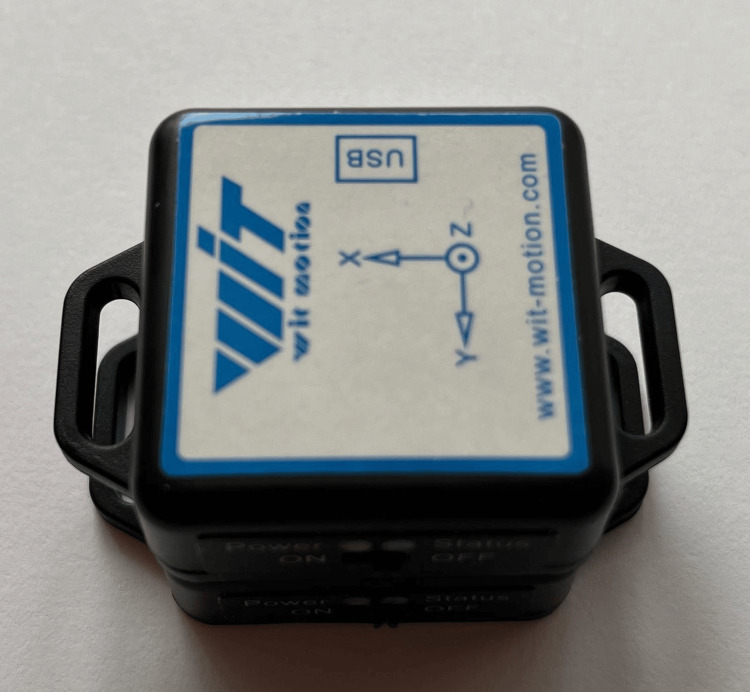
Accelerometer Bluetooth WitMotion sensors

**Figure 2:**
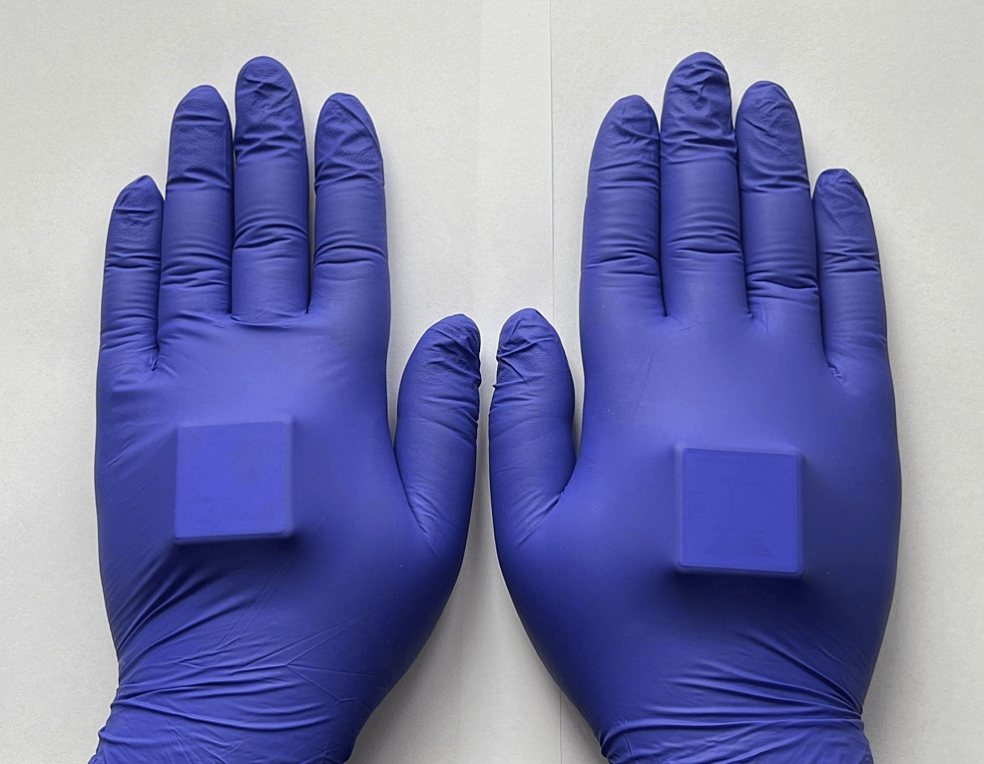
Accelerometer sensors were mounted on the dorsal surface of each hand at the midpoint of the third metacarpal and covered with latex gloves to secure the devices.

A single Accelerometer Bluetooth WitMotion sensor (Figure 1), measuring 4 cm by 4 cm by 1.5 cm and weighing less than 20 grams, was mounted on the dorsal surface of each hand at the midpoint of the third metacarpal. It was covered with latex gloves to help secure the sensor, with the x-axis pointing toward the back of the participant's hand (Figure 2).

These sensors incorporate a high-precision triaxial accelerometer and a triaxial gyroscope that collect the Cartesian coordinates (x, y, z) at a frequency of up to 256 Hz.

Before starting the surgical tasks, the sensors were calibrated in accordance with the manufacturer's instructions to recognize a neutral position and align with specified movement axes.

Data analysis

Objective assessment can be conducted by processing and refining data collected using accelerometers set to a frequency of 128 Hz. This facilitates the evaluation of efficiency parameters such as time, path length, and acceleration.

Time was measured as the total duration needed to complete a surgical maneuver, which directly correlates with the surgeon's experience, and was quantified in seconds (s). Path length represents the total distance that the hand travels during the execution of surgical steps, measured in meters or centimeters (m, cm). Acceleration is defined as the rate at which the hand's velocity changes, measured in meters per second squared (m/s²).

Statistical analysis

The statistical analysis was performed using the Statistical Package for the Social Sciences (SPSS) version 26.0 (Armonk, NY, USA: IBM Corp). Accelerometer data were collected, transmitted to computer software via Bluetooth 2.0, and imported in CSV format. Parameters were calculated using algorithms developed in Python 3.12.2. Each group of surgeons-senior, specialist, and resident-mean values and standard deviations were computed. The Shapiro-Wilk Test assessed the normality of the distribution. The Kruskal-Wallis test was employed to compare procedural time, acceleration, and path length differences across the three surgeon experience levels.

## Results

**Figure 3:**
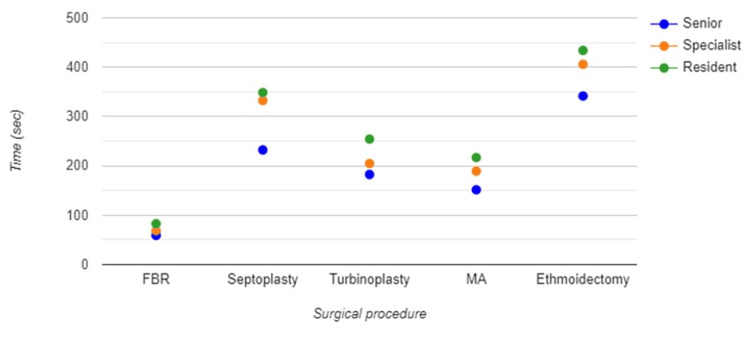
Evaluation and comparison of procedural times The time (seconds) taken to finalize each procedure is shown on the y-axis. Individual procedures are marked on the x-axis. FBR: foreign body removal; MA: maxillary antrostomy Statistical significance appears in all surgical steps (p<0.001) with the biggest difference in the septoplasty group in favor of the senior group.

Although the time taken for the "Foreign Body Removal" procedure does not vary greatly among the different groups, with seniors being the fastest and residents the slowest, there are statistically significant differences between groups (Figure 3).

Compared to other procedures, the time difference is smaller and likely reflects on the simplicity of the procedure, where the level of experience does not significantly impact the time required. All groups appear to perform this relatively simple procedure in a relatively similar timeframe, which may not significantly challenge the group's skill differences (Table 2).

**Table 2:** Representation of mean procedural time for individual surgical steps Mean time to perform the procedure

Procedure	Mean senior time (s)	Mean specialist time (s)	Mean resident time (s)	p-value
Foreign body removal	59	68.6	82.6	<0.001
Septoplasty	232.2	332.5	348.6	<0.001
Endoscopic turbinoplasty	182.5	204.7	254.2	<0.001
Maxillary anthrostomy	151.5	189.3	216.9	<0.001
Ethmoidectomy	341.6	405.9	434	<0.001

There is a notable increase in time, during septoplasty, from seniors to specialists to residents, suggesting that the complexity of this procedure is such that experience significantly impacts performance efficiency. Again, during endoscopic turbinoplasty, seniors are the quickest, followed by specialists, then residents. 

This supports the idea that more experience leads to more efficient procedural times. Septoplasty and turbinoplasty procedures show a clear trend of seniors being faster than specialists, who are, in turn, faster than residents, likely due to the increasing complexity and the requisite skill for efficiency.

The last two more complex procedures, maxillary anthrostomy, and ethmoidectomy, show a wider spread in times, especially for residents, indicating these are likely the procedures where the experience significantly influences the outcome, efficiency, and speed. The increasing procedural time with decreasing experience is consistent across the procedures, further illustrating the learning curve in surgical practice.

**Figure 4:**
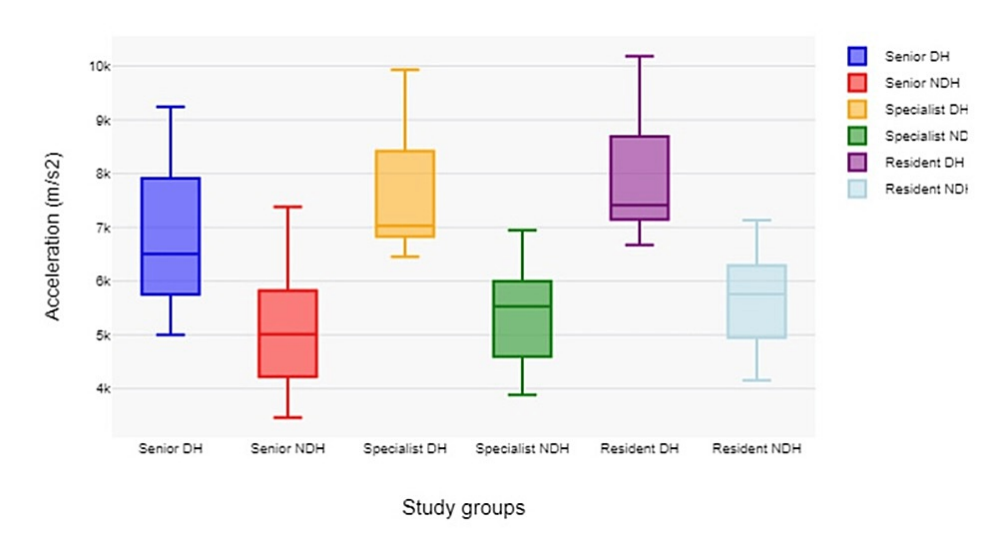
Graphic representation of mean and standard deviation of the resulting acceleration and differences between groups DH: dominant hand; NDH: non-dominant hand. A clear difference can be observed between the resulting acceleration of the dominant hands (instrument hand) and the non-dominant hand (endoscopic hand) as well as between the study groups. The difference between groups reaches statistical significance with a p-value <0.001.

Ethmoidectomy, as a presumably more complex procedure, presents pronounced differences in procedural time, highlighting the impact of experience on the efficiency of performing intricate surgical tasks. The procedural time data complement the previous analysis by confirming that greater experience generally correlates with more efficient, quicker, and more controlled surgical performance. The complexity of the procedures seems to amplify the differences between experience levels, with more complex surgeries showing a greater difference in procedural times across the groups. These insights can help tailor surgical training by focusing on controlling hand movements and improving procedural efficiency, especially for more complex tasks and for less experienced surgeons. Across all procedures, acceleration is generally the highest for the residents' dominant hand, followed by specialists and then seniors (Figure 4).

This suggests that less experienced surgeons make more and faster movements, which may not always translate to efficiency. The non-dominant hand, typically responsible for camera control, shows less variation in acceleration across groups but still follows the trend where residents exhibit the highest values. This could imply that camera handling becomes steadier with experience (Table 3).

**Table 3:** Mean resulting acceleration for all procedures performed by all groups Values are expressed as acceleration (m/s^2^). Higher acceleration is correlated with more uncontrolled movement of the hand and rate of change of speed. DH: Dominant hand; NDH: Non-dominant hand

	Senior		Specialist		Resident		
Procedure	DH	NDH	DH	NDH	DH	NDH	p-value
Foreign body removal	4997.2	7378.3	7027.7	6944.3	7415.8	7136.4	<0.001
Septoplasty	6001.5	5012.2	6452.5	5531.2	6674.1	5757	<0.001
Endoscopic turbinoplasty	7470.4	4471.75	7911.7	4833	8193.7	5208.6	<0.001
Maxillary anthrostomy	6504.1	3458.6	6955.6	3882.3	7308.2	4153.2	<0.001
Ethmoidectomy	9244.7	5307	9932.5	5681.8	10186.3	6011.1	<0.001

The senior group shows less acceleration with the non-dominant hand (NDH) compared to the dominant hand (DH), indicating smoother, steadier movements for camera holding. Specialist and Resident groups also show lower accelerations for the NDH. The specialists' NDH shows relatively little variability in acceleration, possibly indicating a refined technique for camera control. Experienced surgeons (seniors and specialists) tend to have more controlled and efficient movements, reflected in lower mean distances and acceleration, especially with the NDH.

For residents, the larger range and higher median acceleration in the DH might indicate the learning process, where movements are still being optimized for efficiency and precision.

**Figure 5:**
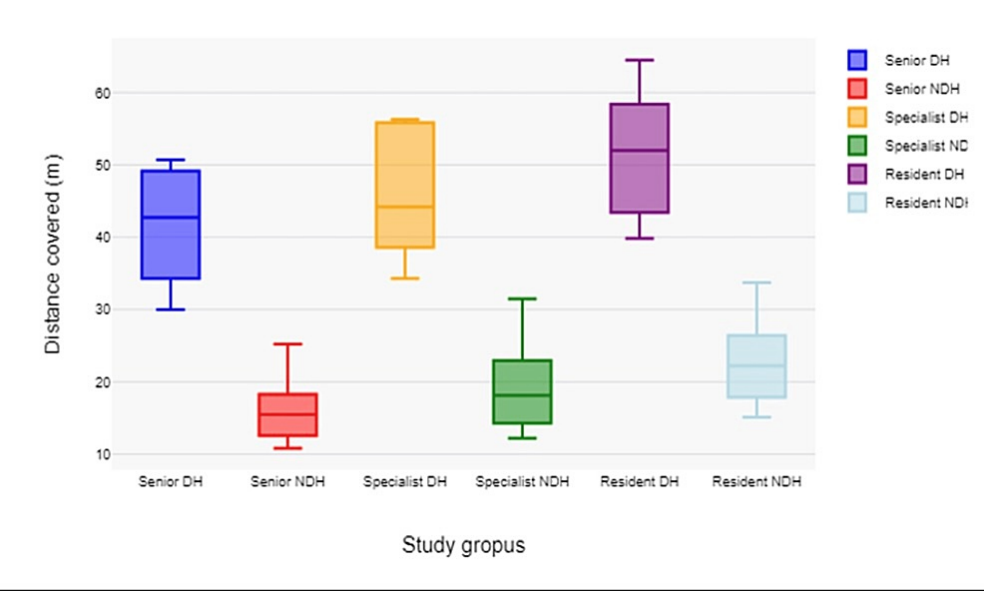
Mean distance covered by participants in all surgical steps DH: dominant hand; NDH: non-dominant hand A statistically significant difference can be seen between the paths covered by each hand of every participant (p<0.001). Also, senior doctors covered significantly less movement with both hands than the specialists and the resident doctors (p<0.001).

The residents' data indicate a steeper learning curve, as seen by higher variability and values in both distance (Figure 5) and acceleration, particularly with the DH, which is used for more complex tasks.

The data reflect role specialization in surgery, with the NDH dedicated to camera control (resulting in steadier, more uniform movements) and the DH performing variable instrument manipulation. The higher values for DH indicate the complexity and dynamic nature of the instrument manipulation tasks compared to the more static role of camera holding (Table 4).

**Table 4:** Mean total distance covered by all participants Values expressed in meters (m) DH: Dominant hand; NDH: Non-dominant hand

	Senior		Specialist		Resident		
Procedure	DH	NDH	DH	NDH	DH	NDH	p Value
Foreign body removal	42.7	10.8	44.2	12.2	52.0	15.1	<0.001
Septoplasty	50.7	16.0	56.3	20.1	64.5	24.0	<0.001
Endoscopic turbinoplasty	35.7	13.2	40.0	15.0	44.6	18.8	<0.001
Maxillary anthrostomy	30.0	15.5	34.3	18.1	39.8	22.2	<0.001
Ethmoidectomy	48.6	25.2	55.7	31.5	56.3	33.7	<0.001

The results could inform training programs to focus on developing steadier hand movements for camera control with the NDH and more efficient, precise movements for surgical tasks with the DH. The senior group's NDH shows significantly lower mean distances covered compared to the DH. This is consistent with the NDH holding the camera, which requires less movement than the instrument-handling DH.

Similarly, both specialists and residents show lower distances covered with the NDH. The residents' DH shows a particularly high mean distance, which might indicate either less efficiency or the need for more movements while learning the techniques. The variability is generally greater for the DH across all groups. This could suggest that the instrument manipulation requires a broader range of movement and possibly more individual variation in how this task is approached.

The path length for the dominant hand mirrors the trend observed in acceleration data, with residents moving the most and seniors the least. The increased path length could be due to less precise movements or more movements necessary to complete the task for those still learning. The non-dominant hand's path length is significantly lower for seniors, suggesting that camera control becomes more efficient with experience.

In our data, the Kruskal-Wallis test has yielded p-values of less than 0.001 for each set of measurements, which is well below the conventional alpha level of 0.05. This means there is a statistically significant difference in procedural time, acceleration, and path length between the groups based on their level of surgical experience. It strongly suggests that the level of training and experience has a measurable effect on both motion metrics and procedural time. The results provide strong evidence to support the conclusion that experience significantly impacts surgical performance metrics.

## Discussion

As expected, seniors generally perform procedures more quickly than specialists and residents, which correlates with the previous analysis showing that seniors have more controlled and efficient hand movements. This efficiency likely contributes to shorter procedural times. Seniors, with their lower and more controlled acceleration, as seen in the previous data, might be using their fine-tuned motor skills to perform procedures more quickly, reflected in their generally lower procedural times.

Specialists appear to take a variable amount of time across procedures, sometimes overlapping with seniors or even residents. This could reflect a phase where specialists are attempting more complex techniques or taking more time to ensure precision, which might be suggested by their higher mean distance and acceleration in hand movements. Specialists who are intermediate in both hand movement and procedural times might be in the process of optimizing their technique, which could sometimes result in longer procedural times due to deliberate practice.

Residents typically take longer to complete procedures, consistent with the higher variability and values in distance and acceleration, indicating that they are still learning and refining their technique. Residents with higher acceleration values might make more rapid movements but lack precision, thus taking longer to complete procedures, aligning with the longer procedural times observed.

The integration of accelerometer-based motion analysis into endoscopic sinus surgery training has the potential to significantly impact the field. Specifically, this approach can provide objective, data-driven insights into surgical skills and techniques, moving beyond subjective assessments; enhance the feedback process by identifying precise movement inefficiencies or errors, allowing for targeted improvements; and accelerate the learning curve for trainees by enabling more efficient and focused skill development. Ultimately, improves patient outcomes by ensuring surgeons acquire the necessary dexterity and precision for safe and effective practice.

Integrating accelerometer-based motion analysis into endoscopic sinus surgery training could revolutionize how surgical skills are taught and assessed. By providing detailed, objective data on hand movements, educators can tailor feedback and training to individual needs, potentially shortening the learning curve and improving surgical outcomes. Forestier et al. demonstrated how motion analysis could identify discriminative patterns of surgical practice, offering a clear pathway toward personalized and efficient surgical training [6].

Other methods for objectively evaluating the learning curve in FESS have been considered. Niederhauser et al. [7] proposed using the analysis of participants' eye movements with eye-tracking glasses as an additional tool for assessing surgical expertise. In eye-tracking, an important measurement is the duration of eye fixations; this refers to the periods when the eyes focus on a specific location. In surgical training, it is used to guide the surgeon's movements and to check the relevant anatomical areas, considering the need for good hand-eye coordination with the 2D endoscopic image. Thus, sixteen residents underwent 18 training sessions using the PHACON Sinus Trainer and demonstrated a significant increase in the percentage of eye fixations on the screen.

In another study [8], an objective assessment of suture placement accuracy, speed, and hand motion efficiency was conducted using a surgical simulator designed for spreader graft placement with cartilage. Twenty-two otolaryngologists, divided into two experience-based groups, used the simulator to position two spreader grafts with three mattress sutures on a model nose employing porcine septal cartilage. An electromagnetic device tracked their hand movements. Metrics such as time to complete the suturing, total hand movement, the number of changes in hand motion direction, and suture placement accuracy were recorded and analyzed for differences between the two groups to validate the simulator's construct. Although both groups showed similar suture precision, the more experienced group performed faster and with fewer hand movements. Participants confirmed the model's realism and educational value through a survey. This suggests that the simulator is an effective tool for objectively assessing and developing suturing skills in cartilage, potentially aiding in the training of surgical residents.

Other authors [9] developed a system for tracking instruments in phonomicrosurgery, featuring a workbench, separate tasks, motion metrics, and a software program. The MicroBIRD tracking system was utilized to monitor the trajectory of surgical instruments' tips within the operative area. Sensors of the microBIRD, which are 0.5 mm in diameter and 3 mm long, were fixed to the instruments' tips to assess the magnitude of the magnetic fields exerted.

Motion data were collected and analyzed from three experts and six beginners. Experts showed significantly smoother movements along the y-axis with their dominant hand. With their non-dominant hand, experts had smoother movements in all directions, shorter travel paths, and improved depth perception (P<0.05). They also achieved a higher quality of operation (P<0.001), although there was no significant difference in time taken (P=5.671). The parameters from this magnetic-based motion tracking system could distinguish between experts and novices, suggesting their potential to enhance phonomicrosurgical training through feedback.

These data underscore the critical role of motion analysis in enhancing surgical training, with accelerometer-based sensors offering a promising avenue for advancing this field, particularly in the context of endoscopic sinus surgery. By addressing the current gaps in surgical skill assessment with innovative technology, this study aims to advance the methodology of surgical training, contributing to the broader goal of elevating surgical care quality.

Additionally, using a sheep's head in the training of endoscopic sinus surgery seems promising, with good partial results already obtained by our team.

An important aspect to mention is that this constitutes the second part of our study; initially, in the first part, we conducted an evaluation of the sheep's head for basic training in functional endoscopic sinus surgery (FESS) with published results [4,5]. Now, we aim to add new elements to our research by objectively assessing the acquisition of surgical skills in FESS by introducing accelerometer sensors into endoscopic surgical training using the ovine model. Thus, we propose the development and implementation of a protocol aimed at improving the learning curve in FESS using a series of efficiency and quality parameters.

To our knowledge, this is the first study that objectively analyses hand motion by using accelerometer sensors attached to the participants' hands during surgical training in FESS, employing the ovine model as an anatomical training model for this type of surgery.

The limitations of our study include a limited number of participants, all from a single training facility. Moreover, the study did not investigate the extent to which the skills acquired using this anatomical model would transfer to actual endoscopic sinus surgeries. Although the ovine model is useful for preparation, it significantly differs from clinical scenarios due to the absence of blood vessels and inflammatory responses in the nasal mucosa commonly observed in surgical practice.

This study opens new possibilities for the evaluation of the ovine model. Therefore, in the future, we aim to assess this training program's effects in determining the minimum number of repetitions required to enhance execution times, acceleration, and the distance covered by both the dominant and non-dominant hands.

Additionally, we wish to compensate for the lack of intraoperative bleeding by translating this assessment to an in vivo ovine model, using general anesthesia with orotracheal intubation, with the assistance of veterinary colleagues, and adhering to all ethical considerations.

## Conclusions

The experience correlates with not only the procedural time but also the motion economy. As surgeons gain experience, they tend to perform surgical tasks with less movement and acceleration, indicating more efficient and controlled procedures.

The data show a clear learning curve from resident to senior, with residents taking longer and moving more to complete the same tasks. Specialists are in the intermediate phase, showing signs of honing their technique towards efficiency. This comprehensive data set can help tailor training programs to focus on both efficiency (quicker procedures) and economy of motion (reduced path length and acceleration), especially in more complex procedures where the difference in performance is more pronounced.

These findings illustrate the significant impact of experience on surgical performance, as seen through the lens of procedural times and quantitative motion analysis. It highlights areas where surgical training can be improved, especially in teaching residents about the economy of motion and procedural efficiency. They underscore the differences in motion between the hands due to their roles and show how experience level affects the motion characteristics in simulated surgical settings.
